# Protective effect of *Silybum marianum* and *Taraxacum officinale* extracts against oxidative kidney injuries induced by carbon tetrachloride in rats

**DOI:** 10.1080/0886022X.2016.1244070

**Published:** 2016-11-15

**Authors:** Ali Karakuş, Yeter Değer, Serkan Yıldırım

**Affiliations:** a Vocational School of Health Services, Hakkari University, Hakkari, Turkey;; b Department of Biochemistry, Faculty of Veterinary Medicine, Yüzüncü Yıl University, Van, Turkey;; c Department of Pathology, Faculty of Veterinary Medicine, Atatürk University, Erzurum, Turkey

**Keywords:** Carbon tetrachloride, kidney, oxidative stress, *Silybum marianum*, *Taraxacum officinale*

## Abstract

The protective effect of the extracts of the plants *Silybum marianum* and *Taraxacum officinale* by carbon tetrachloride (CCl_4_) was researched. Sixty-six female Wistar albino rats were divided into six groups: Control, *Silybum marianum*, *Taraxacum officinale*, CCl_4_, *Silybum marianum*+ CCl_4_, *Taraxacum officinale*+CCl_4_. The *Silybum marianum* and *Taraxacum officinale* extracts were administered as 100 mg/kg/day by gavage. The CCl_4_ was administered as 1.5 mL/kg (i.p.). At the end of the trial period, in the serums obtained from the animals, in the CCl_4_ group it was found that the MDA level increased in the kidney tissue samples as well as in the ALP and GGT enzyme activities. It was also found that the GSH level and the GST enzyme activities decreased (*p*<.05). The microscopic evaluations showed that the CCl_4_ caused a serious hydropic degeneration, coagulation necrosis, and mono-nuclear cell infiltration in the kidney cell. In the animals where CCl_4_ and *Silybum marianum* and *Taraxacum officinale* extracts were applied together, it was found that the serum ALP and GGT enzyme activities decreased and that the MDA level decreased in the kidney tissue, and that the GSH level and GST enzyme activities increased. It was observed that the histopathological changes caused by the CCl_4_ toxicity were corrected by applying the extracts. Eventually, it was determined that the *Silybum marianum* was more effective. *Silybum marianum* and *Taraxacum officinale* extracts which were used against histopathological changes in the kidney caused by toxication showed a corrective effect, which were supported by biochemical parameters.

## Introduction

Kidney is a crucial organ that functions in metabolisms and in the homeostasis of the body. It allows the formation of urine by filtering extra and harmful substances. Nitrogen compounds such as salt, urea, uric acid, and creatinine are also excreted out of the body through urine along with the water.^1^

Carbon tetrachloride (CCl_4_) is widely used in many areas, in particular, in the dry cleaning industry as an industrial solvent. CCl_4_ is metabolized by mitochondrial monooxygenase (P450 2E1) in the liver. The free radicals appearing in the meantime pave the way for cell damage, causing peroxidation of fatty acids in the phospholipids in the membranes of the cells.^2–5^

It has been determined that the target organ of CCl_4_ which could be taken in the body via respiration, digestion, and the skin is not only the liver. The kidney is reported to cause the formation of free radicals by spreading into other tissues like the kidney, heart, lung, testis, brain, muscle, and blood.^6–10^ It has also been stated that exposure to this solvent causes acute or chronic renal failure.^11^^,^^12^

The specific tests for the diagnosis and prognosis of the intoxication occurred in the body as a result of chemical effects like CCl_4_ and monitoring the response of the therapy applied measures the activities of alkaline phosphatase (ALP), gamma glutamate transferase (GGT), alanine aminotransferase (ALT), aspartate aminotransferase (AST), and lactate dehydrogenase (LDH) enzymes.^13^^,^^14^

In the determination of renal toxicities (Na), potassium (K), chlorine (Cl), calcium (Ca), phosphate (P) should be measured as serum electrolytes; however, the levels of creatinine, urea, and uric acid should be measured as kidney function indices.^15^

Malondialdehyde (MDA) occurs as a final product of lipid peroxidation (LPO) that free radicals induce. The MDA leads to negative consequences such as changes in ion permeability in cell membranes and enzyme activity.^16^

Organisms, under normal physiological conditions, have free radicals formed by endogenous or exogenous causes and the antioxidant defense system that grows depending upon them struggles with oxidative stress. In particular, glutathione peroxidases (GPX), glutathione S-transferase (GST), and glutathione reductase (GR) enzymes which are associated with glutathione (GSH) metabolism play an important role in this system.^16^

With the increasing importance of phytotherapy in today's modern medicine, medicinal plants are often used in order to support the body’s antioxidant mechanisms and for protection against the diseases and disorders that free radicals may generate.^17^^,^^18^ The researches have shown that antioxidative activity of medicinal plants is rather high.^19^^,^^20^

Plants such as *Silybum marianum* (L.) Gaertner, *Cynarascolymus* L., *Taraxacum officinale* Weber, and *Angelica archangelica* L. containing phytochemicals are utilized for both prevention and treatment in liver and kidney diseases, either individually or in combination.^16^^,^^21^

*Silybum marianum* L. (thistle) has often been used in the treatment of digestive system problems and liver diseases since ancient times.^22–24^*Silybum marianum* has an antioxidant property owing to the flavonolignans and other polyphenolic compounds in it and it, accordingly, has the function of keeping free radicals.^25^

The most important components of the root of *Taraxacum officinale* Weber (dandelion) are inulins, lactones, triterpenes, sterols, flavonoids, and phenolic acids, and it has antioxidant property thanks to these substances.^26^

In this study, the protective effects of plant extracts of *Silybum marianum* and *Taraxacum officinale*, whose antioxidant properties against oxidative injury induced by CC1_4_ have already been proven, on the kidney are aimed to be searched comparatively.

## Materials and methods

Wistar albino female rats (*n* = 66, 200–250 g) were used in the study. During the 20-day trial period, they were maintained in the cages in the temperature controlled rooms (22 ± 2 °C) with a 12-h light/dark cycle. The study was carried out in accordance with the article 10/03.10.2013 of the Animal Experiments Ethics Committee.

They were given standard food pellet and drinking water *ad libitum*. The rats used in the study were randomly chosen and were divided into six groups as: Control (C), CCl_4_ (C), *Silybum Marianum* (SM), *Taraxacum Officinale* (TO), *Silybum Marianum*+ CCl_4_ (SM + C), *Taraxacum Officinale*+ CCl_4_ (TO + C).

**Group 1: Control group (K, *n* = 7):** Received standard rat chow and drinking water.

**Group 2: Silybum marianum group(SM, *n* = 7):** Received *Silybum marianum* extract (Solgar, Leonia, NJ), dissolved in water, at a dose of 100 mg/kg/day orally for 20 days.

**Group 3: *Taraxacum officinale* group (TO, *n* = 7):** Received *Taraxacum officinale* extract (Solgar, Leonia, NJ), dissolved in water, at a dose of 100 mg/kg/day orally for 20 days.

**Group 4: CCl_4_ group (C, *n* = 15):** Injected with a single dose of 1/1 suspension CCl_4_ with olive oil at a dose of 1.5 mL/kg/day intraperitoneally (i.p.) in order to obtain liver toxicity.

**Group 5: *Silybum marianum* + CCl_4_ group (SM + C, *n* = 15):** were given *Silybum marianum* extract (Solgar, Leonia, NJ), dissolved in water, at a dose of 100/mg/kg/day for 30 days. Toxicity was induced by treating them with a single dose of CCl_4_ olive oil suspension mixture of 1/1 at the dose of 1.5 mL/kg/day intraperitoneally (i.p.).

**Group 6: *Taraxacum officinale* + CCl_4_ group (TO + C, *n* = 15):** were given *Taraxacum officinale* extract (Solgar, Leonia, NJ), dissolved in water was given at a dose of 100 mg/kg/day for 30 days. Toxicity was induced by treating them with a single dose of CCl_4_ olive oil suspension mixture of 1/1 at the dose of 1.5 mL/kg/day intraperitoneally (i.p.).

In order to strengthen the antioxidant system of the animals in groups 5 and 6, *Silybum marianum* and *Taraxacum officinale* extracts were started to be administered 10 days before they were given CCl_4_.

At the end of the trial period, the hearts of the rats were directly cannulated after the application of 75 mg/kg ketamine (i.p.) and their blood samples were placed in the anticoagulant vacuum tubes. Their serum was separated after the centrifugation at room temperature (+4 °C) and at 3000 rpm for five minutes. Alkaline phosphatase, GGT enzyme activities and urea, uric acid, creatinine, Na, K, Ca, Cl, and P levels which were extracted from the serum were measured in the autoanalyzer (Cobas Integra 800) using commercial kit Roche/Hitachi, Basel, Switzerland.

After their water was removed between two pieces of filter paper, the extracted kidney tissues were weighed and then stored in a deep freezer (−20 °C) until the analyses were conducted. The kidney tissue was homogenized in RIPA buffer for measuring the MDA level. GSH level and GPx, GR, GST enzyme activities were homogenized by adding cold phosphate buffer (pH 6–7). The homogenates obtained were centrifuged at room temperature (+4 °C) at 10,000 rpm for 15 min. The supernatants formed after the centrifugation were measured in ELISA device (Zenyth 200 rt) using a commercial kit (Cayman Chemical Company, Ann Arbor, MI).

The kidney tissue samples taken for histopathological observation were fixed in 10% formalin solution for 48 h and then were washed in running tap water for 8 h. After they were treated with alcohol (70°, 80°, 90°, 96°, and 100°) and a series of xylene during the routine tissue control period, they were blocked in paraffin. The samples were prepared on the slides by taking 4 μm-thick cuts from each block. They, which were prepared for the histopathological examination, were stained with hematoxylin–eosin (HE) and the relevant areas were photographed, analyzing with light microscope.

Appropriateness of continuous variables to normal distribution was analyzed with Kolmogorov–Smirnov test. For the variables showing normal distribution, one-way analysis of variation (one-way ANOVA) was used for the comparison of the groups. *p*<.05 was considered as the level of significance in the analyses. All the analyses were made using SPSS (20.0) software (SPSS Inc., Chicago, IL).

## Results

**Figure 1.**
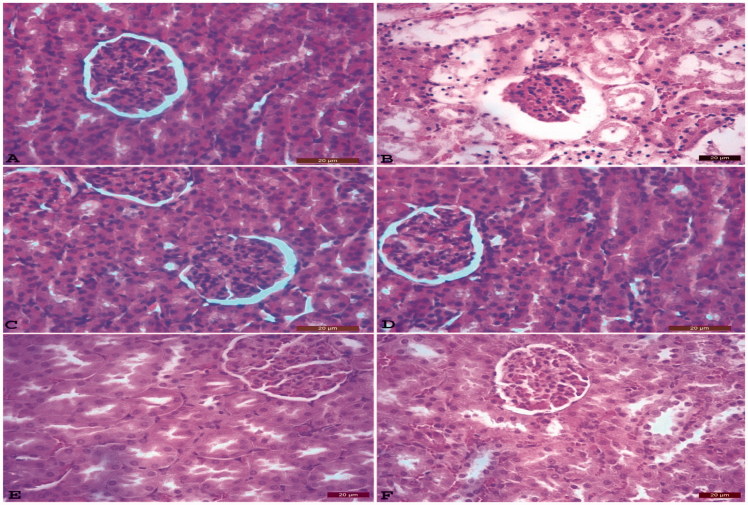
The normal histopathological structure of kidney tissue of the control (K) group (A). Atrophy in the glomerulus, dilation in Bowman's capsule and tubulus, severe hydropic degeneration of tubular epithelium, coagulation necrosis, tubule epithelium scattered on tubular lumen histopathological structure of kidney tissue of the CCl_4_ (C) group (B). Histopathological structure of kidney tissue of *Silybum marianum* (SM) group (C). Histopathological structure of kidney tissue of *Taraxacum officinale* (TO) group (D). *Silybum marianum*+ CCl_4_ (SM + C) group: kidneys, hyperemia, and degeneration in very few numbers of tubular epithelium (E). *Taraxacum officinale*+ CCl_4_ (TO + C) group: mild hydropic degeneration of tubular epithelium, very few numbers of necrotic cells (F) HxE, Bar: 20 μm.

At the end of the study, ALP, GGT enzyme activities; the levels of urea, uric acid and creatinine, the levels of Na, K, Ca, Cl, and P are shown in Tables 1 and 2 successively. MDA and GSH levels and GPx, GR, and GST enzyme activities are shown in Table 3. Histopathological changes in the kidney tissue samples from all groups are presented in Figure 1.

**Table 1.** Serum ALT, GGT enzyme activities and urea, uric acid, creatinine levels of the control and trial groups.

Groups	ALP (U/L)	GGT (U/L)	Urea (μmol/L)	Uric acid (mg/dL)	Creatinine (μmol/L)
K	73.48 ± 3.47^c^	1.23±.05^c^	40.73 ± 3.03^c^	1.08±.12^c^	0.90±.07^b^
SM	76.53 ± 6.08^c^	1.17±.09^c^	41.96 ± 3.27^c^	1.00±.1^c^	0.94±.06^b^
TO	75.45 ± 5.27^c^	1.26±.06^c^	41.70 ± 2.97^c^	1.05±.06^c^	0.96±.09^b^
C	168.98 ± 13.25^a^	3.88±.44^a^	129.3 ± 8.11^a^	4.01±.26^a^	1.65±.08^a^
SM + C	131.85 ± 7.26^b^	2.61±.36^b^	80.43 ± 6.76^b^	2.68±.21^b^	1.44±.06^a^
TO + C	132.53 ± 10.39^b^	2.80±.32^ba^	82.65 ± 7.36^b^	2.82±.14^b^	1.44±.09^a^

a,b,c The difference between the group averages with different letters in the same column is statistically significant, *p*<.05.

Data represent the mean ± SD of rats.

**Table 2.** Na, K, Ca, Cl, and P levels of control and trial groups.

Groups	Na (mmol/L)	K (mmol/L)	Ca (mmol/L)	Cl (mmol/L)	P (mmol/L)
K	142.28 ± 4.47^a^	143.38 ± 2.28^a^	10.81±.23^b^	95.33 ± 0.49^c^	5.93 ± 0.08^c^
SM	144.66 ± 6.16^a^	142.28 ± 4.47^a^	10.72±.43^ba^	95.5 ± 0.76^c^	6.05 ± 0.07^cb^
TO	144.46 ± 3.86^a^	145.13 ± 3.40^a^	10.05±.28^ba^	93.83 ± 1.16^c^	6.05 ± 0.07^cb^
C	166.78 ± 4.52^a^	140.14 ± 2.30^a^	12.51±.93^a^	112.66 ± 1.33^a^	6.43 ± 0.069^a^
SM + C	155.18 ± 6.42^a^	142.28 ± 4.32^a^	11.81±.52^ba^	102.16 ± 0.94^b^	6.30 ± 0.07^ba^
TO + C	155.36 ± 4.47^a^	144.22 ± 2.12^a^	11.43±.55^ba^	100.08 ± 0.73^b^	6.36 ± 0.1^ba^

a,b,c The difference between the group averages with different letters in the same column is statistically significant, *p*<.05.

Data represent the mean ± SD of rats.

**Table 3.** MDA and GSH levels of kidney tissue and GP_X_, GST, GR enzyme activity levels of control and trial groups.

Groups	MDA (μM)	GSH (μM)	GP_X_ (nmol/min/mL)	GST (nmol/min/m)	GR (nmol/min/mL)
K	0.77 ± 0.2^d^	13.43 ± 0.53^a^	35.25 ± 2.12^a^	77 ± 6.13^a^	148.60 ± 10.43^ba^
SM	0.73 ± 0.27^d^	13.73 ± 0.55^a^	37.83 ± 3.36^a^	75.83 ± 6.95^ba^	155.73 ± 3.91^a^
TO	0.75 ± 0.17^d^	12.95 ± 0.45^a^	34.84 ± 4.23^a^	78.66 ± 6.86^a^	154.05 ± 4.29^a^
C	1.75 ± 0.4^a^	8.84 ± 0.39^b^	22.85 ± 3.03^a^	34.91 ± 6.46^c^	84.93 ± 7.84^c^
SM + C	1.33 ± 0.27^b^	10.38 ± 0.63^b^	26.66 ± 3.06^a^	54.08 ± 7.10^ba^	115.85 ± 10.31^cb^
TO + C	1.48 ± 0.17^c^	9.33 ± 0.55^b^	26.50 ± 4.67^a^	49.16 ± 3.79^ba^	107.05 ± 9.91^c^

a,b,c,d The difference between the group averages with different letters in the same column is statistically significant, *p*<.05.

Data represent the mean ± SD of rats.

## Discussion

Carbon tetrachloride is a colorless and volatile substance that can get into the body via respiration, digestion and skin, and it is used in experimental animals due to its toxic effect.^27^ The toxic effect of CCl_4_ may damage liver cells and other tissue cells and membranes through LPO of oxy and hydroxy radicals and other ways has been experimentally shown in *in vivo*/*in vitro* environments.^5^^,^^9^^,^^10^^,^^28^

In the study conducted, they determined that in the rats in which they induced liver and kidney toxicity using CCl_4_, serum ALP, ALT, AST, and LDH enzyme activities as well as the levels of urea, uric acid, creatinine, Na, K, Ca, MDA increased significantly and the GSH level and SOD activity increased as well. They stated that this was caused by the liver and kidney histopathology and the breakdown of their functions.^29^

In their study, using CCl_4_, found that serum ALT, AST, bilirubin, and creatinine levels elevated remarkably; however, GSH level and SOD activity decreased, MDA level increased and severe hydropic degeneration formed in nephron epithelium in the kidney tissue. They also detected that serum ALT, AST, bilirubin, and creatinine levels decreased; serum ALT, AST, bilirubin, creatinine levels decreased; kidney tissue GSH level and SOD activity increased, and MDA level and severe hydropic degeneration in nephron epithelium decreased after Alafia multiflora root methanol extract had been administered. They stated that this could have occurred because of the protective effect of Alafia multiflora root extract against the CCl_4_ induced damage in the rats.^30^

In the conducted studies on rats to investigate the toxic effects of melamine they observed that serum urea, uric acid, creatinine, K, P, Ca levels, which are important for kidney and ion metabolism, elevated, yet Na and Cl levels decreased in the groups melamine was given. They stated that the results were due to the formation of toxicity occurred in the kidney.^15^

In another study, reported that serum ALT, AST, AGT, ALP enzyme activities and urea, uric acid, creatinine, Na, K, Ca, kidney tissue MDA levels significantly increased, tubular occlusion formed and epithelium scattered on and tubulus lumen were seen in the group CCl_4_ was administered. The reason for this, they said, was the structure of the liver and kidney and the impairment of their functions. They observed that this activity and the levels decreased, and tubular occlusion and epithelium scattered on and tubulus lumen decreased as well in the rat groups that were treated with honey and silymarin. They said that this resulted from the fact that honey and silymarin had flavonoid and phenolic compounds that show antioxidant properties.^31^

They observed elevation in serum ALT, AST enzyme activities and a decrease in GP_X_, CAT, SOD activities besides albumin levels in the rat groups in which CCl_4_ was administered. However, they noticed a decrease in serum ALT, AST enzyme activities and an increase in GP_X_, CAT, SOD enzyme activities and albumin levels in the groups ginger was given. As a result, they concluded that ginger herb could be used as a preventive substance for the body.^32^

Xylaria nigripes (XN-T), which is widely used in traditional Chinese medicine, was given to the rats in which they induced toxicity via CCl_4_ administration. They observed that while there was a decrease in serum ALT, AST enzyme activities and liver tissue SOD, CAT, GPX activities and an increase in MDA level in the group CCl_4_ was administered, there was an increase in serum ALT and AST enzyme activities and liver tissue SOD, CAT, and GPX activities besides a decrease in MDA level in the group XN-T was given. They concluded that this effect resulted from the pressure of XN-T, which is a powerful antioxidant, on oxidative stress.^28^

In their study,^33^ they administered CCl_4_ i.p. for four weeks in order to form toxicity. While an increase in MDA, H_2_O_2_, NO_2_ levels in the kidney tissues was observed, they detected a decrease in CAT, SOD, GSH, GST, GPX, and GR activities in the groups that received CCl_4_. However, while there was a decrease in MDA, H_2_O_2_, NO_2_ levels in the kidney, they noticed an increase in CAT, SOD, GSH, GST, GPX, and GR activities in the groups that *Sonchus asper* was administered. They concluded that these changes increased because of the fact that CCl_4_ increased the oxidant systems, suppressing the antioxidant systems and that *Sonchus asper* pressurized the oxidative stress.

In the rats they formed toxicity using CCl_4_,^34^ administered melatonin to the rats. They observed a decrease in kidney tissue SOD, GSH-Px, CAT, GST activities and GSH levels, an increase in MDA level, coagulation necrosis, glomerular atrophy, and interstitial edema. However, they determined an increase in kidney tissue SOD, GSH-Px, CAT, GST activities, and GSH levels but a decrease in MDA level besides improvement in the histopathology in the groups melatonin was administered. They stated that this effect was resulted from the melatonin which has an antioxidant feature and pressurized the oxidative stress.

Induced toxicity in the rats by applying CCl_4_ and searched the protective role of *Bridelia retusa S***.** rind extract. As a result of the histological studies of the rats, after the kidney tissue toxicity was formed, they observed dilation in the bowman capsule and scattered glomerular structure. However, they reported that it was effective in the improvement of damaged histopathological structure after the therapeutic application of the extract.^35^

In the present study, it was determined that in the group CCl_4_ was administered—compared to the control group—serum ALP, enzyme activities, urea, uric acid, creatinine, sodium, potassium, calcium, chlorine, phosphate, and kidney tissue MDA levels increased; GSH level and GP_X_, GST, GR enzyme activities decreased; serum ALP, GGT enzyme activities, urea, uric acid, creatinine, Na, K, Ca, Cl, P, and kidney tissue MDS level decreased in the groups *Silybum marianum* (SM) vs *Taraxacum officinale* (TO) extracts were given with CCl_4_ as a protective substance; GSH level and GP_X_, GST, GR activities increased and, finally, these changes were more in the SM + CCl_4_ group.

In conclusion, it was determined that *Silybum marianum* (SM) vs *Taraxacum officinale* (TO) plant extracts lowered serum ALP, enzyme activities, urea, uric acid, creatinine, Na, K, Ca, Cl, P, and kidney tissue MDA level in the protection against oxidative injury induced by CCl_4_ in kidney damage; increased GSH level and enzyme activities related to GSH metabolism and these changes were supported by histopathological findings. It was also found that SM was more effective in this situation.

It was additionally concluded that the plant extracts which were used against histopathological changes occurring in the kidney as a result of toxicity showed a corrective effect and this was supported by the biochemically observed parameters; *Silybum marianum* and *Taraxacum officinale* extracts could be used as protective and supportive of treatment for endogenous and/or exogenous-originated kidney toxicity.
